# Progressive Retinal Toxicity Nine Years After Hydroxychloroquine Discontinuation: A Case Report

**DOI:** 10.7759/cureus.98674

**Published:** 2025-12-07

**Authors:** Christina Karakosta, Susan M Downes

**Affiliations:** 1 Ophthalmology, Oxford University Hospitals NHS Foundation Trust, Oxford, GBR; 2 Oxford Eye Hospital, Oxford University Hospitals NHS Foundation Trust, Oxford, GBR; 3 Nuffield Laboratory of Ophthalmology, University of Oxford, Oxford, GBR

**Keywords:** hydroxychloroquine, retinal toxicity, rheumatoid arthitis, screening, secondary retinopathy

## Abstract

We report a case of hydroxychloroquine (HCQ) retinal toxicity showing continuous progression over nine years after cessation of HCQ. A 55-year-old female had a history of rheumatoid arthritis treated with HCQ 200 mg daily for 20 years (cumulative dose ≈1.46 kg). She had no previous retinal screening and was asymptomatic. She was referred in 2016 for the first time, and the drug was discontinued following detection of characteristic parafoveal retinal changes on spectral-domain optical coherence tomography and fundus autofluorescence consistent with HCQ toxicity. Despite discontinuation, serial imaging revealed ongoing progression of outer retinal and retinal pigment epithelium atrophy over nine years, with gradual enlargement of the parafoveal lesion and progression of the paracentral scotoma in both eyes. Best-corrected visual acuity remained stable in both eyes. This case highlights that retinal damage from HCQ may progress for many years after cessation, emphasizing the importance of early detection to reduce the risk of progression in this irreversible condition. Review post-cessation may identify those in whom further progression has occurred, who may benefit from practical support, such as visual aids and or assistive technology.

## Introduction

Hydroxychloroquine (HCQ) is widely used for autoimmune diseases such as systemic lupus erythematosus and rheumatoid arthritis due to its favorable systemic safety profile [1]. However, retinal toxicity, albeit rare, remains a serious, dose-dependent adverse effect [2]. Traditionally, the risk increases significantly after five years of use or cumulative doses exceeding 1 kg [3].

HCQ binds to melanin in the retinal pigment epithelium (RPE), leading to impaired lysosomal function, photoreceptor degeneration, and RPE atrophy [3]. Although toxicity is generally considered to stabilize after discontinuation, recent evidence suggests that progression may continue for years [4]. Here, we report a case of ongoing retinal degeneration nine years after discontinuation of HCQ, despite cessation upon early detection of toxicity.

## Case presentation

A 55-year-old woman with a 20-year history of rheumatoid arthritis was referred to our department for HCQ retinal toxicity screening. She had been taking HCQ 200 mg daily for 20 years (estimated cumulative dose = 1.46 kg, average body weight = 60 kg). She had no previous screening and was asymptomatic. She was referred to our department in 2016 for the first time, and HCQ was immediately discontinued after the detection of parafoveal outer retinal loss and juxtafoveal hyperautofluorescence area on fundus autofluorescence (FAF), consistent with HCQ toxicity.

**Figure 1:**
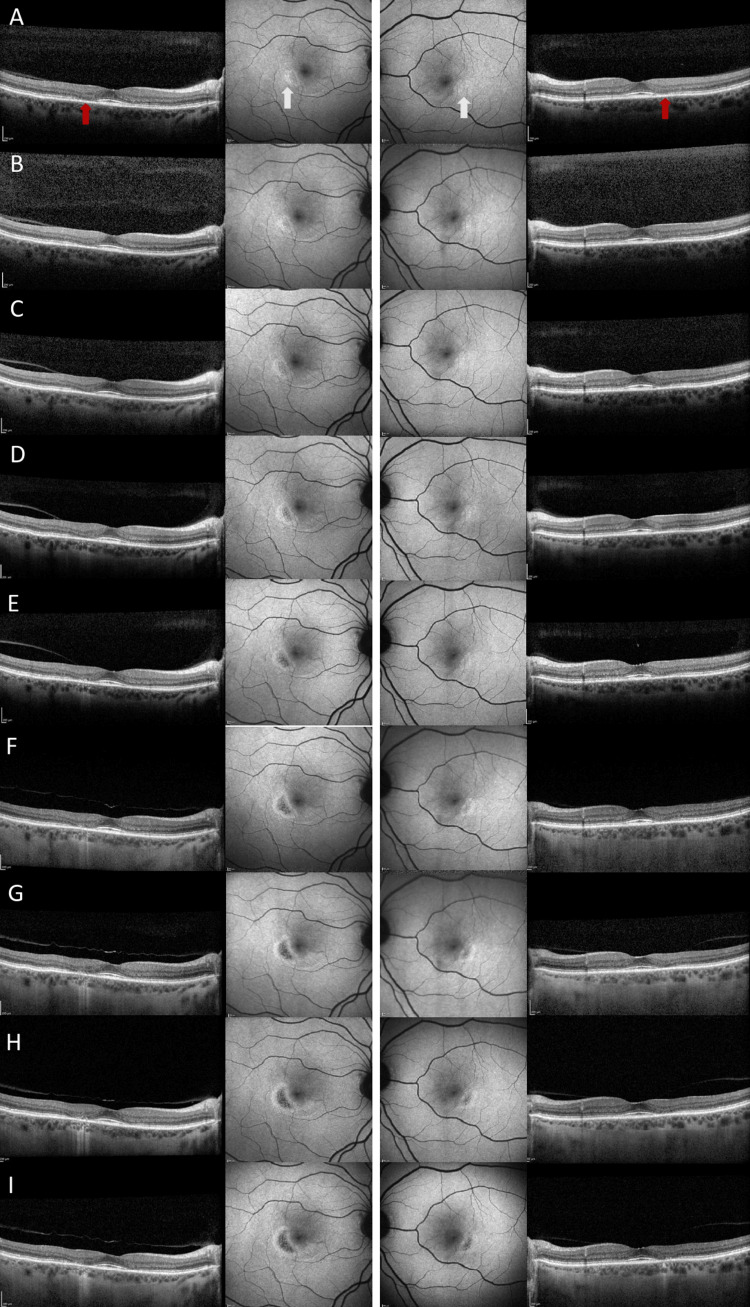
Optical coherence tomography (OCT) and fundus autofluorescence (FAF) images of the right and left eye over a nine-year period. Panel A shows baseline imaging, and Panels B–I represent annual follow-up images from year two through year nine. FAF images are displayed in the central columns, with OCT scans in the outer right and outer left columns. Red and white arrows are displayed in Panel A to identify the baseline parafoveal ellipsoid zone disruption on OCT and the crescent-shaped hyperfluorescent area on FAF, respectively. The same anatomical regions are demonstrated in Panels B–I as they progress over time.

At the time of discontinuation, best-corrected visual acuity (BCVA) was 0.1 in the right (OD) and left eye (OS). Spectral-domain optical coherence tomography (SD-OCT) revealed parafoveal ellipsoid zone disruption with preserved foveal contour (“flying saucer” sign). FAF demonstrated a symmetrical crescent-shaped hyperfluorescent area inferotemporal to the fovea in both eyes (Figure 1).

**Figure 2:**
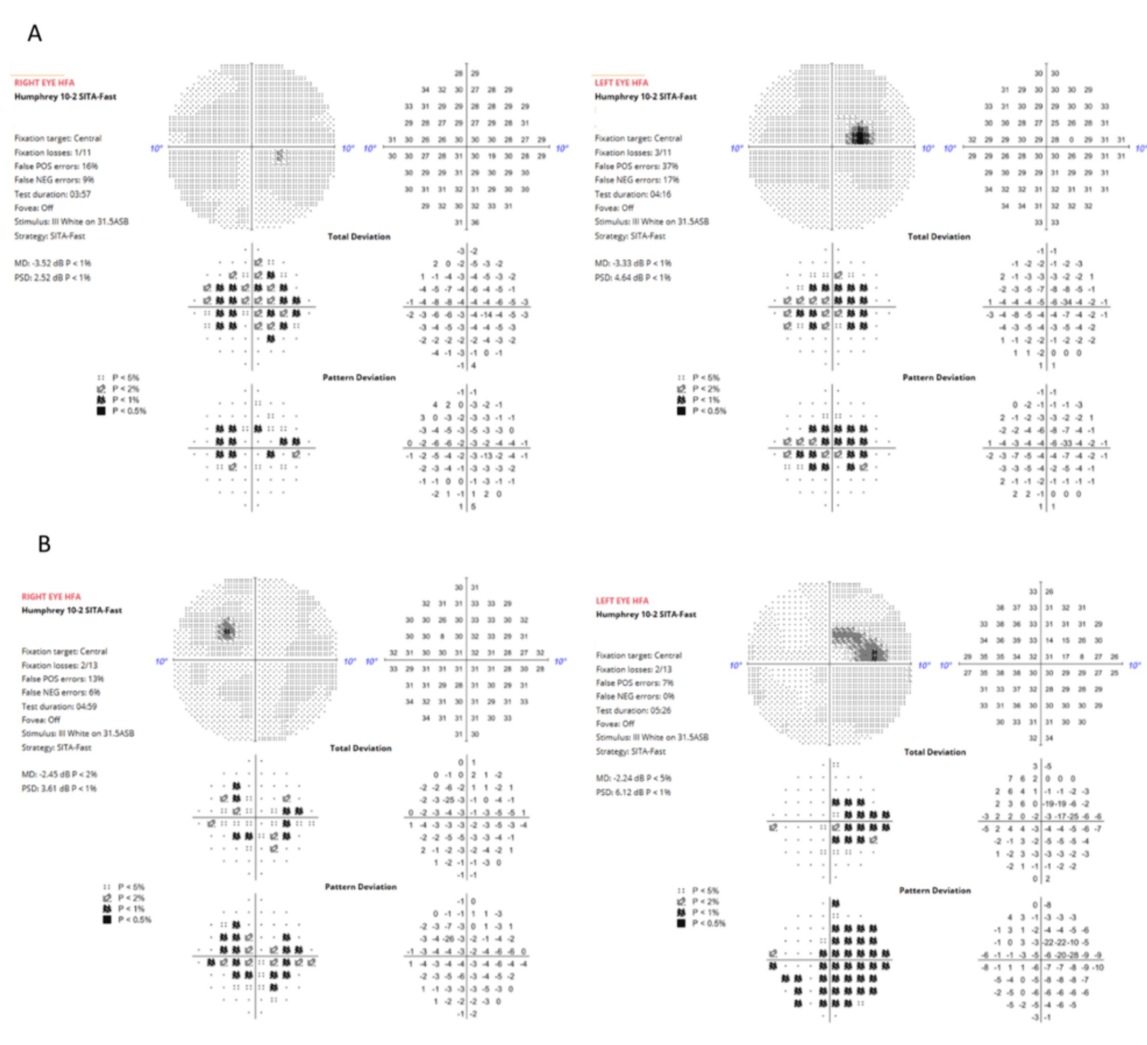
Humphrey visual field test 10-2 in 2019 (A) and 2025 (B) showing the progression of the paracentral scotoma in both eyes.

Despite discontinuation, over the last few years, the patient had started to notice a patch in her vision, particularly in the right eye. Annual follow-up imaging revealed continued expansion of the parafoveal atrophic zone and progressive ellipsoid zone loss (Figure 1). By 2025, nine years post-discontinuation, BCVA had remained stable (0.12 in OD and 0.12 OS), but the paracentral scotoma had progressed in both eyes (Figure 2).

FAF showed a crescent-shaped hypofluorescent area inferotemporal to the fovea surrounded by hyperfluorescence (Figure 1).

No alternative etiology for retinal degeneration was identified, including age-related macular degeneration or inherited dystrophy. Genetic testing for inherited retinal dystrophies was performed and yielded negative results. Systemic evaluation remained stable.

## Discussion

This case demonstrates that retinal damage can continue for nearly a decade after drug withdrawal, even when toxicity was identified early, and the cumulative dose was within acceptable limits relative to body weight.

Epidemiology, risk factors, and screening practices

The cumulative dose, duration of therapy, and daily dose by real body weight are the principal determinants of HCQ toxicity risk. Long-term exposure beyond 5-10 years substantially increases prevalence, even within the recommended dosing limits [5-7]. Population-level studies demonstrate that real-world screening coverage remains inconsistent, with a significant proportion of long-term users never undergoing guideline-based retinal monitoring [5]. These gaps highlight the need for systematic screening programs and reinforce the American Academy of Ophthalmology recommendation of baseline and annual multimodal testing after five years of use.

Imaging biomarkers and phenotypic spectrum

Advances in multimodal imaging have revolutionized HCQ retinopathy detection. SD-OCT and FAF are now central for identifying early parafoveal and pericentral structural changes [3,8,9]. Yusuf et al. described characteristic OCT features such as ellipsoid-zone disruption and parafoveal thinning, while FAF reveals the classic “bull’s-eye” pattern and expanding zones of hypoautofluorescence [3,8]. These modalities can reveal subclinical damage even in patients with normal visual fields, improving early recognition. Our patient’s serial OCT and FAF imaging, which documented progressive outer retinal and RPE atrophy despite cessation, mirror the findings described in these studies.

Vascular changes and advanced imaging

Newer reports employing swept-source OCT angiography (OCTA) have revealed reduced choriocapillaris flow and deep capillary plexus attenuation in eyes with HCQ toxicity, suggesting that vascular compromise may accompany or even precede structural loss [10-12]. Quantitative OCTA analysis has correlated choriocapillaris perfusion deficits with the extent of photoreceptor damage, proposing a dual mechanism of direct photoreceptor-RPE toxicity and secondary ischemic injury [12]. These observations provide a pathophysiologic rationale for continued degeneration after drug withdrawal, as ongoing microvascular dysfunction might perpetuate damage even in the absence of circulating HCQ.

Disease progression after cessation

Emerging literature highlights the heterogeneity in outcomes after cessation; some patients show stabilization, while others exhibit a slow but steady increase in the area of atrophy for years [9]. Several longitudinal and retrospective studies have documented that HCQ retinopathy can progress long after discontinuation [13-16]. Pham et al. demonstrated that the rate of progression correlates with the stage of retinopathy at cessation; eyes with early-stage disease often stabilize, while those with advanced outer-retinal/RPE atrophy show continued expansion of lesions [4].

Case series with long follow-up similarly describe progressive ellipsoid-zone loss for up to four years post-withdrawal [15]. Potential modifiers under active investigation include baseline severity at the time of cessation, cumulative exposure, comorbid retinal disease, and genetic susceptibility [3,9]. In our patient, comprehensive genetic testing for inherited retinal dystrophy was negative, supporting this “post-cessation progression” phenotype and reducing the likelihood that an occult hereditary disorder explains the delayed progression [3,9]. Given the documented variability in post-cessation course, these studies collectively support our clinical approach of long-term imaging surveillance and cautious functional prognosis counseling. The advent of artificial intelligence to aid detection of retinal structural changes is expected to enhance an early pick up of HCQ toxicity [17].

Genetic and individual susceptibility

Growing interest surrounds the possibility that genetic predisposition contributes to HCQ toxicity. Zaidi et al. reviewed evidence for genetic factors and noted ethnic variation in prevalence, although no single high-penetrance mutation has been validated [18]. More recent genomic and pharmacogenomic studies suggest potential associations between variants in genes related to lysosomal and autophagy pathways and susceptibility to HCQ-induced retinal damage [19]. However, results remain exploratory, and further research is needed. In our case, comprehensive genetic testing for inherited retinal dystrophies was negative, reducing the likelihood of a coexisting monogenic retinal dystrophy and strengthening the attribution of progression to drug toxicity rather than inherited disease.

Emerging concepts and future directions

Recent reviews emphasize that HCQ retinopathy should be viewed as a spectrum rather than a static endpoint, with ongoing cellular injury possible even after drug cessation [11,13]. Proposed mechanisms include persistent intracellular accumulation of HCQ in melanin-containing RPE cells, lysosomal dysfunction, oxidative stress, and impaired phagocytosis leading to photoreceptor apoptosis [20]. Microvascular and inflammatory contributions are also increasingly recognized [12]. Regular monitoring after discontinuation has been advocated, with annual or biennial OCT and FAF imaging and consideration of OCTA to detect subtle changes in choriocapillaris perfusion [5,10,12].

## Conclusions

HCQ retinopathy may continue to progress long after cessation of therapy, particularly in patients with advanced retinal changes at the time of discontinuation. Prolonged cumulative exposure and pre-existing outer retinal or RPE damage appear to increase the likelihood of ongoing degeneration. Our patient’s course illustrates this phenomenon, with a clearly documented progression of atrophy that was ongoing nine years after cessation of therapy. Patients should be counseled about the potential for delayed progression and maintain long-term surveillance with multimodal imaging. Early detection, individualized dosing, and continuous monitoring remain essential to minimizing irreversible visual loss associated with HCQ toxicity.
